# Affordance Boundaries Are Defined by Dynamic Capabilities of Parkour Athletes in Dropping from Various Heights

**DOI:** 10.3389/fpsyg.2017.01571

**Published:** 2017-09-20

**Authors:** James L. Croft, John E. A. Bertram

**Affiliations:** ^1^Centre of Exercise and Sports Science Research, School of Medical and Health Sciences, Edith Cowan University Perth, WA, Australia; ^2^Biomedical Engineering, University of Calgary Calgary, AB, Canada; ^3^Cumming School of Medicine, University of Calgary Calgary, AB, Canada

**Keywords:** affordances, transitions, movement patterns, landing mechanics, momentum

## Abstract

Available behaviors are determined by the fit between features of the individual and reciprocal features of the environment. Beyond some critical boundary certain behaviors become impossible causing sudden transitions from one movement pattern to another. Parkour athletes have developed multiple movement patterns to deal with their momentum during landing. We were interested in whether drop distance would cause a sudden transition between a two-footed (precision) landing and a load-distributing roll and whether the transition height could be predicted by dynamic and geometric characteristics of individual subjects. Kinematics and ground reaction forces were measured as Parkour athletes stepped off a box from heights that were incrementally increased or decreased from 0.6 to 2.3 m. Individuals were more likely to roll from higher drops; those with greater body mass and less explosive leg power, were more likely to transition to a roll landing at a lower height. At some height a two-footed landing is no longer feasible but for some athletes this height was well within the maximum drop height used in this study. During low drops the primary task constraint of managing momentum could be achieved with either a precision landing or a roll. This meant that participants were free to select their preferred landing strategy, which was only partially influenced by the physical demands of the task. However, athletes with greater leg power appeared capable of managing impulse absorption through a leg mediated strategy up to a greater drop height.

## Introduction

Choosing appropriate movements requires that we perceive how features of the environment relate to our action capabilities, or what they afford (Gibson, 1977). Action capabilities are partly determined by a person's size, such as height or limb length. People are able to perceive boundaries to actions based on the relationship between these geometric properties and reciprocal properties of the environment. For example, the perceived boundary for climbing stairs is partly explained by the ratio of leg length to riser height (Warren, 1984). Similarly, body-scaled affordances can be perceived for passing-through apertures of certain width (Warren and Whang, 1987), reaching objects at certain distances (Mark, 1987; Carello et al., 1989; Bootsma et al., 1992; Rochat and Wraga, 1997; Wagman and Day, 2014), and grasping objects of certain lengths (Cesari and Newell, 1999; Richardson et al., 2007).

However, as Chemero (2003) points out, body scale may be a convenient surrogate for ability, but it is rarely a good one. Indeed, maximum stair height to leg length ratio differs for different populations: less in the elderly (Konczak et al., 1992) and greater in females with high joint flexibility (Meeuwsen, 1991), suggesting that action capabilities also depend on non-geometric factors such as strength and joint flexibility. The relationship between these individually perceived dynamic capabilities and features of the environment that provide opportunity for particular kinds of behavior are called action-scaled affordances (Fajen et al., 2009) and have been shown to constrain perception of affordances for behaviors such as jumping (Pepping and Li, 2000; Ramenzoni et al., 2008). Most affordances are some combination of body-scaled and action-scaled (Fajen et al., 2009). Defining geometric features is relatively intuitive but defining which dynamic capabilities of the perceiver are important for reciprocal features of the environment requires a deeper understanding of the task. We have previously noted that even for a common behavior such as locomotion the functional task can be obscure, highlighting the importance of properly defining and understanding the task being undertaken (Croft et al., in press).

From a dynamical systems perspective, once the affordance boundary has been reached a spontaneous transition occurs to another mode of behavior. There are many situations in which different movement patterns/strategies can be utilized for a similar general goal but where each is most advantageous under a given set of circumstances. Walking at slower speeds and running at faster speeds is an example. Over some range of intermediate speeds both are potential choices. For such cases a discrete transition event is observed (a gait change) when it is perceived that the advantage of one gait supersedes the other. The transition can have some ambiguities, however. For instance, the speed at which gait transitions occur can be different for incremental speed increases from slow walking vs. incremental speed decreases from a fast running speed (Thorstensson and Roberthson, 1987). Transitions similar to the walk-run and run-walk are also observed in discrete tasks. For example, as riser height increases in a set of stairs, at some height it is unnatural to step up in the usual manner, and indeed a transition to a different movement occurs (Konczak et al., 1992).

To investigate these non-linear transitions in dynamic activities, we sought a task that could rely on geometric and dynamic features of the performer where they have more than one possible mode of behavior. The urban sport Parkour often involves high velocity impacts against solid surfaces. For example, Parkour athletes routinely drop from obstacles of various heights, reportedly sometimes over 20 feet (6.1 m). They have learned different landing techniques to manage the impacts—a two-foot (precision) landing, and a roll. Which geometric and dynamic features of the performer define the affordance boundary for dropping and landing safely using a two-foot landing?

Actions such as stepping off an object subject the body to gravitational accelerations and the ability to accomplish the landing task safely depends on limiting the impact force to tolerable levels. As the height of the object increases so does landing velocity, and as a consequence the individual's momentum prior to landing (P = m • v). The primary task of landing is to dissipate the momentum in a manner in which maximum load (f), loading velocity (or the related differential of loading velocity, jerk), or the accumulated energy level does not exceed biological limits (e.g., muscle tear or tendon rupture). The magnitude of momentum can be changed by applying an impulse (J) over some time (t), where J = ∫ f dt. Strategies that increase the time over which the impulse is spread decrease force magnitude. For instance, allowing the supporting joints to flex while loaded to moderate levels can gradually decrease momentum with modest force. Alternatively, it is possible to redirect the force vector, such as converting translational momentum into rotational momentum with a roll, so that force becomes oriented in a direction that does least harm (as in the classic parachutist's roll). The strategies that are available to an individual to achieve this task vary based on their organismic constraints (e.g., height, weight, bone, joint and muscle strength, flexibility, and coordination). If the chosen strategy is insufficient to manage the pre-contact momentum musculo-skeletal injury will result.

Being able to use different movement strategies to reach the same performance outcomes reflects the degeneracy of biological systems (Whitacre, 2010), specifically defined as “the ability of elements that are structurally different to perform the same function or yield the same output” (Edelman and Gally, 2001, p. 13763). Secondary task constraints, such as the amount of horizontal momentum present before the drop, or the subsequent direction of travel will also influence the selection of an appropriate movement. As task constraints change, skilled performers can take advantage of degenerate solutions—solutions that they are aware of and are able to choose because of previous experience—to adapt to the specific situation (Seifert et al., 2013). Parkour athletes have learned to satisfy the primary task goal of dissipating landing momentum by using two main techniques: a precision landing on the “forefoot or balls of the feet, bending the knees to absorb impact”, or a shoulder roll “in the direction of travel, leading with one side of the body and finishing on the opposite side of the body” (Puddle and Maulder, 2013, p. 123), i.e., rolling on a diagonal axis, not like a gymnastics roll about a medial-lateral axis.

If rolling is selected to deal with large pre-landing momentum it is reasonable that at some drop height individuals will switch from a precision landing technique, to a roll technique, as per their Parkour training and/or their physical limitations. Sudden transitions from one movement pattern to another have been observed in other discrete actions (e.g., stair climbing, boxing, lifting) and in cyclical actions (e.g., walk-run transition) that occur due to changes in a control parameter. For example, when moving an object between two shelves the height of the lower shelf from the floor determines whether people choose a squat technique or a stoop technique (Burgess-Limerick et al., 2001). The purpose of this research was to investigate whether similar transitions in movement pattern (i.e., precision landing or a roll) could be elicited based on varying the control parameter (i.e., drop height). We were further interested in which variable(s) triggered the transition between movement patterns, whether related to kinetics, kinematics, or organismic factors (body mass, segment lengths, maximum vertical jump ability).

## Methods

### Participants

Nine male and two female individuals aged 18–32 years, of height 1.58–1.87 m and mass 54–92 kg volunteered for this study. Each had at least 2 years of experience in Parkour. Individuals gave written informed consent to take part in the study that was approved by the local ethical review board.

### Measures

Prior to testing, anthropometric measures (standing height, hip height, and body mass) were taken. Following a sports specific warm-up, counter-movement jump height was measured using a Vertec (Vertec, Sports Imports, Hilliard, OH) with the best of three jumps used for analysis. Ground reaction forces were measured using force plates (9287CA and 9287BA, Kistler, Switzerland) and each individual's movement pattern during the landing was recorded in three dimensions using a 10-camera motion capture system (V-MX-F20, Vicon, UK). Retro-reflective rubber markers were used to define an eight segment model (thorax, pelvis, thighs, shanks, and feet). Subjects were told to ignore the markers and act as though they normally would. Kinematic (i.e., movement) data was be recorded at 200 Hz and kinetic (i.e., force) data at 1,000 Hz.

### Procedure

Individuals were required to step off a box of varying height. The initial box height of 0.60 m was increased in a fixed sequence by 15–25 cm^1^ until a maximum of 2.30 m, or until the individual indicated that the box was higher than they would usually jump from. The height was decreased in a similar manner back to the initial height and then increased to the maximum height once more giving a maximum of 27 trials. We gave minimal instructions to the subjects to ensure the task represented real-world activities so the participants would spontaneously choose their own movement pattern—they were simply told to behave as they would during a typical Parkour setting. The landing surface was Mondo Super X (Mondo, Italy) over concrete.

### Data processing

Kinematic data was reconstructed in Vicon Nexus (v2.1, Vicon, UK) using standard procedures and exported with the kinetic data to Matlab (R2016a, Mathworks, Natick, Mass, USA) for further processing. Center of pressure was filtered with a zero-lag fourth order lowpass Butterworth filter with 25 Hz cut-off.

The start of the impulse was identified by finding the first instance that vertical ground reaction force exceeded 200 N and then stepping backwards in time iteratively until force no longer exceeded four standard deviations of baseline noise above the baseline mean (similar to Liebermann, 2008). Pre-landing momentum was calculated from the velocity of the hip marker immediately before the start of the impulse, and the mass of the individual. The duration required for the ground reaction force to return to body weight was also determined. Leg stiffness was calculated directly from kinematic–kinetic measures (Coleman et al., 2012) by calculating the component of force acting to compress the leg as if it were a spring (from the center of pressure to the greater trochanter position in the sagittal plane).

### Statistical analysis

Of primary interest was the ability to predict the type of landing strategy based on the box height, anthropometric measures and power measures. Following Kruschke (2014) we avoided null hypothesis statistical testing, NHST, and analyzed the data with Bayesian logistic regression. “Unlike traditional NHST-based statistics, Bayesian analysis yields complete distributional information regarding the parameters in the regression model. Bayesian analysis uses only the observed data and does not use *p*-values and confidence intervals that are based on hypothetical unobserved data that might have been obtained assuming a particular stopping intention about sample size of the researcher.” (Kruschke, 2014, pp. 738–739).

We used a logistic regression model, in which a roll (*y*_*i*_ = 1) on the *i*^th^ trial is described by a logistic function with a linear combination of predictor values:

$$\begin{array}{l} \mu_{i}=logistic(\beta_{0}+\;\beta_{1}x_{1i}+\;\beta_{2}x_{2i}+\;\beta_{3}x_{3i\;}+\;\beta_{4}x_{4i}\;)\\ y_{i}\;\sim \;Bernoulli\;(\mu_{i}) \end{array}$$

where

$$logistic\left(x\right)=\frac{1}{\left(1+\exp\left(-x\right)\right)}$$

and *x*_1_ is box height, *x*_2_ is the individual's mass, *x*_3_ is individual's leg length, and *x*_4_ is their counter movement jump height (maximum of three trials). We used non-committal broad priors^2^ on the parameters so that the prior had minimal influence on the posterior.

The posterior distribution was generated as a Markov Chain Monte Carlo (MCMC) sample using the free software R, rjags, and JAGS (Plummer, 2003). We followed typical steps for this type of analysis: Three chains were burned in (for 1,000 steps) and a total of 300,000 steps were saved; autocorrelation and mixing were checked graphically and confirmed numerically with Gelman-Brooks-Rubin diagnostics—there was little to no variation between the chains, so the resulting MCMC sample is highly representative of the underlying posterior distribution.

Similar techniques have been used extensively in other fields and their use is increasing in psychological science (van de Schoot et al., 2017) and similar Bayesian logistic regressions have been used to determine changes in behavior in pedestrian collision avoidance (Croft and Panchuk, in press).

## Results

**Figure 1:**
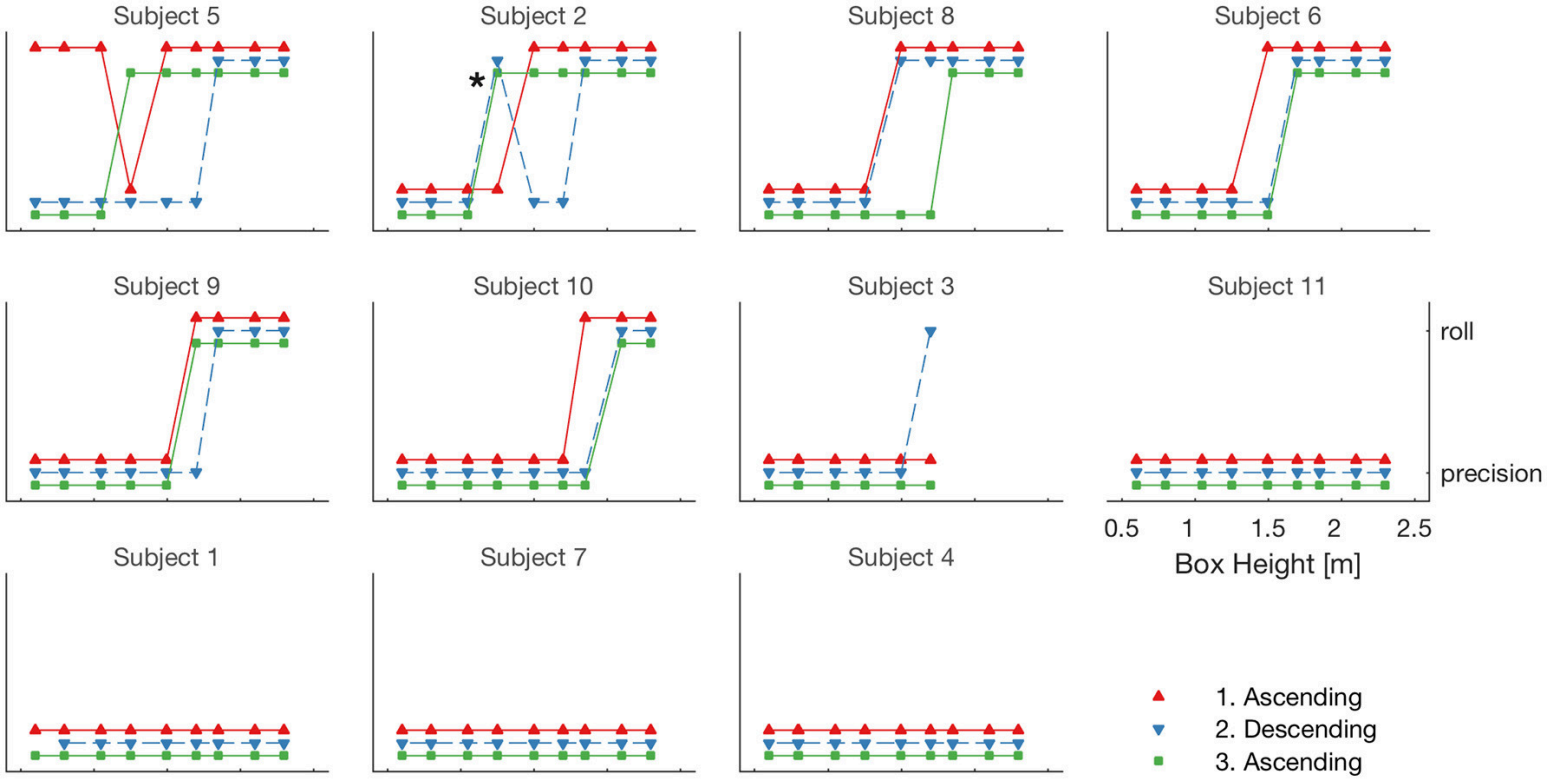
Each panel corresponds to a subject; the order of the panels is determined by the height where that subject first rolled. For each subject the box height increased, decreased, and increased again. Each of these series are indicated by different color and symbol type. For each height the movement pattern (precision landing or roll) is plotted. The ^*^ indicates a roll from a moderately low height and is discussed in the text.

Ten of the eleven subjects completed all 27 trials; one indicated they would not usually jump off walls higher than 1.7 m (subject 3) and so only completed 18 trials. Four subjects did not employ the roll strategy at any height (subjects 1, 4, 7, and 11), one subject (subject 3) rolled only once (from the highest box height achieved), and the remainder used a combination of precision landings and rolls (see Figure 1). The predicted transition from precision to roll with increasing height occurred in four individuals (subjects 6, 8, 9, and 10). Subject 2 followed a similar trend except for one trial where he chose to roll from a low height (indicated in Figure 1). Subject 5 sometimes rolled from very low heights and performed precision landings from much greater heights.

**Figure 2:**
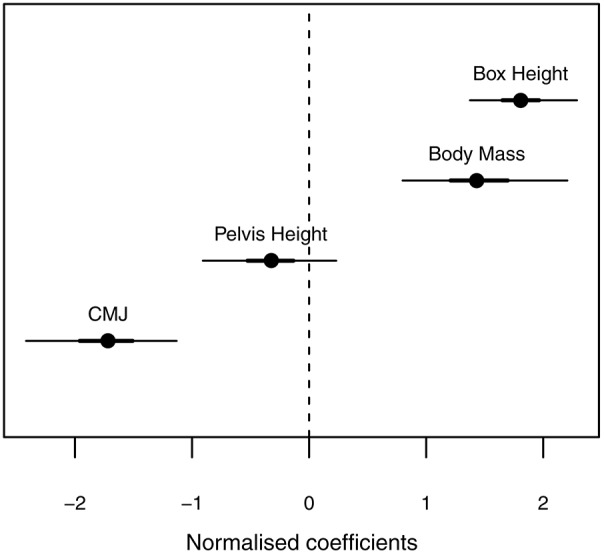
Credible intervals for the predictor coefficients of the posterior distribution. Thick lines show the 90% highest density intervals (HDI) and thin lines show the 95% HDI. Drop height, body mass, and countermovement jump height predict the likelihood of rolling. Drop height and body mass have a positive correlation with rolling—higher drops and higher body mass are more likely to roll; countermovement jump height has a negative correlation with rolling—subjects with a lower countermovement jump performance is more likely to roll at a given height.

Our primary aim was to assess whether the transition between movement patterns, was related to organismic factors (body mass, leg length, and leg power—counter-movement jump height) and task constraint (drop height). Body mass and drop height directly affect pre-contact momentum, leg length affects the distance over which the impulse can be applied. Results from the logistic regression model (Figure 2) indicated that the likelihood of a roll was predicted by drop height (individuals were more likely to roll from higher drops), body mass (heavier individuals were more likely to roll), and countermovement jump height (those with a lower jump height were more likely to roll). Leg length did not predict the likelihood of rolling.

**Figure 3:**
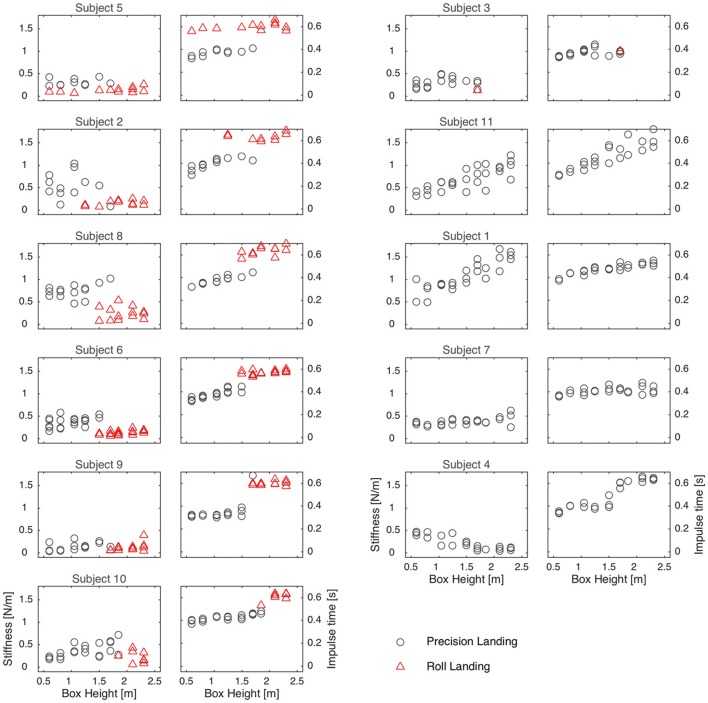
Each pair of panels corresponds to a subject; the order of the panels is determined by the height where that subject first rolled, as in Figure 1. For each box height the stiffness (left panel of pair) and impulse time (right panel of pair) are plotted. Precision landings are plotted in gray circles and rolls in red triangles.

So what caused these athletes to switch from a precision landing to a roll? Leg stiffness and impulse time followed a similar trend with increasing drop height for those subjects that did roll at some height (subjects 2, 5, 6, 8, 9, and 10). As drop height increased, impulse time generally increased and stiffness remained approximately constant (through visual inspection of Figure 3). When they rolled, the impulse time was greater and leg stiffness decreased or stayed the same. Of those individuals that did not roll (subjects 1, 4, 7, and 11; and 3, who only rolled once) there were two different trends: stiffness and impulse time increased linearly with drop height for subject 1 and subject 11; but for subject 4 and subject 7, (and to some extent subject 3) stiffness remained low regardless of drop height and impulse duration was generally long.

## Discussion

In the current experiment, we attempted to stimulate similar transitions in movement pattern (i.e., precision landing or a roll) based on varying the control parameter (i.e., drop height). Within certain limitations we found results consistent with a transition event. Why didn't we observe perfect relationship between box height and movement pattern? Firstly, for safety reasons we only used box heights up to 2.3 m, which was not high enough to require a roll movement from all individuals. This is akin to increasing stair riser height but stopping before some very long-legged or flexible individuals transitioned to another movement pattern (or even those trying to “show off” their ability to manage extreme situations). The second reason is related to the availability of each movement in each situation. The movement that is most suitable for low stairs is not possible for very high stairs, the movement that is suitable for slow locomotion (walking) is not possible for fast locomotion, and the movement that is suitable for low drops (precision landing) is not feasible for very high drops—neither of the two precision landing strategies (high leg stiffness and tolerance of high force magnitude and low leg stiffness and accommodation of momentum through increase in impulse duration) likely remain possible for drops from great heights.

However, the converse is not true. It is possible to climb up a very low step, run at very slow speeds, and roll from a small drop. We do not usually choose these options because it is not energetically advantageous to do so (e.g., Bertram, 2015). However, in the same way that children's play is not driven by energetic optima, Parkour athletes derive enjoyment from interacting physically with their environment. These individuals do not need to roll from lower heights but some of them enjoy doing so (possibly enhanced in the lab setting where the floor surface was rubberized). This can be seen in Figure 1 where subject 5 rolled at modest heights, used precision landings at higher heights in the same series, and precision landings at low heights in another series. So from very high drops (albeit higher than used in this study) it is likely these athletes would always roll, but at lower heights they have the choice of either movement. A similar observation has been reported in children playing on different height blocks arranged at varying distances apart (Prieske et al., 2015), where children chose to jump down between blocks even when stepping was possible. Drawing from the work of Sheets-Johnstone (2003) the authors suggest the children experienced joy through the lively action of jumping compared to the less dynamic action of stepping. In the same vain, we suggest that rolling is more fun than a precision landing, “because it resonates in feelings of aliveness radiating dynamically through a kinetic/tactile-kinesthetic body” (Sheets-Johnstone, 2003, p. 417).

Given the degenerate solutions available: how do these athletes choose the most appropriate movement for a given set of constraints? The primary task of landing is to dissipate the momentum in a manner to avoid injury but when this can be satisfied in multiple ways then presumably secondary constraints influence the selection of movement, whether related to an intrinsic desire to have fun, or to convert momentum to the direction of future travel. As Richardson commented, “whether an affordance *can* be actualized in a certain manner does not always correspond with whether an individual *will* actualize an affordance in that way” (Richardson et al., 2007, p. 856).

At the outset we had predicted that organismic factors, such as body mass or leg length, may be involved in determining the transition between available movement patterns, i.e., the affordance provided by the box was both geometry- and action-scaled. If the primary task is to dissipate pre-contact momentum it seems reasonable that any factors that contribute to increases in pre-contact momentum may influence the choice of landing strategy. Since momentum is defined by body mass and contact velocity, it seemed reasonable that heavier athletes may transition at lower drop heights due to their greater momentum—this was supported by the model.

Leg length affects the distance over which the impulse can be applied. So individuals with longer legs should be able to absorb more pre-contact velocity in a precision landing, thereby delaying their transition to a roll. However, we did not find evidence to support this. There was a substantial range of leg lengths in our sample (pelvis heights varied from 0.84 to 1.13 m) but possibly it had such a minor role that its effect is hidden by other constraints. There is a possibility that the small effect of leg length is masked by another variable, but leg length and body mass were only mildly correlated (*R*^2^ = 0.42), suggesting that there was enough independent variation to detect their individual relationships with the outcome variable.

We also predicted that athletes with greater explosive power, as measured by countermovement jump performance (Markovic et al., 2004), would transition from a precision landing to a roll at higher drop heights, and this was supported by the results. An explosive jump requires the generation of large forces, indicating high strength levels in the muscle. Although a jump depends on positive work generation while a drop landing requires eccentric loading of muscle, it is likely that greater overall muscle strength allows the eccentric tolerance of greater force as well. Even though the legs of these athletes would be subjected to higher force levels, greater muscle strength likely allows them to control and tolerate precision landings from higher drop heights. Possibly, by developing explosive power Parkour athletes could increase the height at which precision landings could be tolerated.

The experiment was set up to observe how these athletes chose to land in a task that represented one they may experience during their urban sport. We gave minimal instructions to the subjects to ensure the task represented real-world activities so the participants would spontaneously choose their own movement pattern. More clearly defined task goals, such as maximizing efficiency or minimizing risk of damage, may have resulted in more consistent transitions, or we could have asked them to land in a precision landing whenever possible and only use a roll when necessary to avoid injury. However, this is akin to examining a walk-run transition by asking people to walk until they really can't walk anymore and only then transition to a run. This is not how humans act outside of a lab; they make choices based on energy efficiency, comfort, perceived risk or other less readily quantified factors such as personal satisfaction (fun).

## Ethics statement

This study was carried out in accordance with the recommendations of Human Research Ethics Committee of Edith Cowan University with written informed consent from all subjects. All subjects gave written informed consent in accordance with the Declaration of Helsinki. The protocol was approved by the Human Research Ethics Committee of Edith Cowan University.

## Author contributions

Conception (JC, JB); acquisition, analysis (JC); draft and revision (JC, JB); final approval of the version to be published (JC, JB); agreement to be accountable for all aspects of the work (JC, JB).

### Conflict of interest statement

The authors declare that the research was conducted in the absence of any commercial or financial relationships that could be construed as a potential conflict of interest.

## Footnotes

^1^The increment differed slightly because we had boxes of 20 and 45 cm. Different combinations created unequal increments but the same sequence (0.60, 0.80, 1.05, 1.25, 1.50, 1.70, 1.85, 2.10, 2.30 m) was used for all individuals.

^2^β_0_ ~ *T*(0, 0.1, 1), β_1−4_ ~ *T*(0, 0.25, 1)
